# Prophylaxis with oral zinc sulfate against radiation-induced oropharyngeal mucositis in patients with head and neck cancer: Protocol for a meta-analysis of randomized controlled trials

**DOI:** 10.1097/MD.0000000000013310

**Published:** 2018-11-30

**Authors:** Ting Shuai, Li-Juan Yi, Xu Tian, Wei-Qing Chen, Hui Chen, Xiu-E Li

**Affiliations:** a Department of Nursing, Peking University of Stomatology Hospital, Beijing; b Department of Nursing, Hunan Traditional Chinese Medical College, Zhuzhou; c Chongqing Key Laboratory of Translational Research for Cancer Metastasis and Individualized Treatment, Chongqing University Cancer Hospital & Chongqing Cancer Institute and Chongqing Cancer Hospital, Chongqing, China.

**Keywords:** head and neck cancer, oropharyngeal mucositis, radiotherapy, systematic review, zinc sulfate

## Abstract

**Background:**

Oropharyngeal mucositis is considered to be an inevitable and the most troubling side effect of head and neck irradiation, which is caused by the direct toxic action of radiation on oral mucosa. Several randomized controlled trials (RCTs) have investigated the efficacy of zinc sulfate in prevention of radiation-induced oropharyngeal mucositis in patients with head and neck cancer. However, the definite conclusions have not been confirmed. The systematic review and meta-analysis will be performed to comprehensively evaluate whether zinc sulfate is effective in prevention of radiation-induced oropharyngeal mucositis in patients with head and neck cancer.

**Methods:**

Relevant information will be identified by a comprehensive search of the following electronic databases: PubMed, Cochrane Central Register of Controlled Trials (CENTRAL), Embase, and EBSCO from their inception to October 2018. Only RCTs which evaluated whether zinc sulfate is effective in prevention of radiation-induced oropharyngeal mucositis in patients with head and neck cancer will be eligible. Two independent investigators will be assigned to search literature, screen information, extract data, and appraise the risk of bias. The primary investigator will use Revman 5.3 software to perform all statistical analyses. We designed this systematic review and meta-analysis on June 5. 2018, and expect to complete the full-text on December 20, 2018.

**Results:** This protocol has been registered with a unique identifier of CRD42018108533. We will submit this systematic review and meta-analysis to a peer-reviewed journal. Meanwhile, we also will dissemination the whole findings in topic-related conferences.

**Conclusion:** As the first systematic review and metaanalysis, the present study will elucidate current evidence of the role of oral zinc for the prevention and treatment of radiation-induced oropharyngeal mucositis in the head and neck cancer.

## Abstract 2

Supplemental Digital Content is available in the text

## 1 Introduction

Head and neck cancers are a related group of cancers involving the oral cavity, pharynx (oropharynx, nasopharynx, hypopharynx), and larynx.^[1]^ With the rapid aging and growth of the population worldwide, head and neck cancer incidences are rapidly growing. According to the newest data reported in Global Cancer Statistics 2018, head and neck cancer has been the 8th most common cancer and the number of newly diagnosed cases of these neoplasms is about 710 thousand in 2018.^[2]^ The prevalent method of radical head and neck cancer treatment is radiotherapy used alone or in combination with chemotherapy or surgery.^[3,4]^ However, radiotherapy with or without chemotherapy can cause some troublesome side effects.^[5]^ Among these side effects, oropharyngeal mucositis is considered to be an inevitable and the most troubling side effect of head and neck irradiation, which is caused by the direct toxic action of radiation or chemotherapeutic agents on oral mucosa.^[6,7]^ It is reported that approximately 60% of patients receiving radiotherapy of the head and neck cancer can develop oropharyngeal mucositis and the rate will increase to 90% if chemotherapy is concurrently given.^[8]^ Oropharyngeal mucositis has 4 presentations including initial erythema, subsequent ulceration, necrosis, and bleeding.^[9]^ Local pain, eating and swallowing difficulties with nutritional problems, and speech problem is the other symptoms of oropharyngeal mucositis. These uncomfortable symptoms can worsen the patient's quality of life.^[10,11]^ Moreover, when oral mucosal effects get severe, the radiotherapy program need to be changed such as delays in administration or limitation in radiation dosage, which may weaken the radiotherapy effects and decrease the survival of patients in the head and neck cancer.^[12–14]^ Therefore, it is an important objective to prevent or minimize oropharyngeal mucositis as much as possible.

At present, some ways in prophylaxis and treatment of oropharyngeal mucositis related to radiation therapy are developed, such as low-level laser therapy and several organic products. But the effects of these interventions have not yet been completely confirmed.^[15,16]^ Hence, choosing the appropriate way to prevent cancer therapy-induced oropharyngeal mucositis is still necessary to go on exploring. Evidence suggests that zinc sulfate is beneficial against oxidant damage and the progression of reactive oxygen species (ROS)-induced disease.^[17,18]^ And the efficacy of zinc sulfate is to scavenge rapidly superoxide radicals from the environment and accelerate the activity of erythrocyte Cu-Zn SOD enzyme.^[19]^ To date, there are limited trials that evaluate and show the benefit of zinc sulfate in reducing radiation-induced mucositis in the head and neck cancer.^[9,19–21]^ Of these studies, 3 studies showed zinc could reduce the incidence and severity of mucositis in cancer patients undergoing radiotherapy with or without chemotherapy.^[9,20,22]^ Gorgu et al^[21]^ found that zinc sulfate prophylaxis did not reduce the incidence of mucositis. So the efficacy of zinc to prevent radiation-induced oropharyngeal mucositis remains controversial.

Hence, the purpose of the present study is to perform the systematic review and meta-analysis to comprehensively evaluate whether zinc sulfate is effective in prevention of radiation-induced oropharyngeal mucositis in patients with head and neck cancer.

## 2 Methods

**Figure 1:**
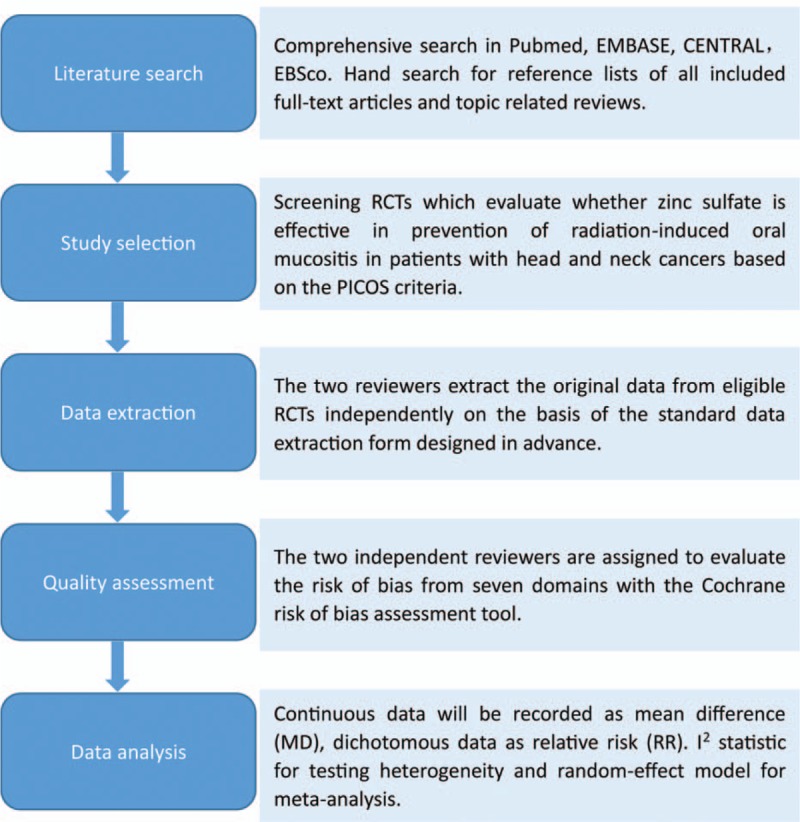
The process of the whole study.

The meta-analysis has been registered in the International Prospective Register of Systematic Reviews (PROSPERO) platform with the unique identifier of CRD42018108533. The protocol is designed based on the Preferred Reporting Items for Systematic Reviews and Meta-Analysis Protocols (PRISMA-P) 2015: elaboration and explanation.^[22]^ All statistical analyses in the meta-analysis will be implemented according to the recommendations proposed by Cochrane Collaboration (CC).^[23]^ The process of the whole study is summarized in Figure 1. We designed this systematic review and meta-analysis on June 5, 2018, and expect to complete the full-text on December 20, 2018.

### 2.1 Selection criteria

The selection criteria are established according to *PICOS* acronym: Participants (P): Individuals is older than or equal 18 years with histologically proven cancers of the head and neck. They are in good performance status with a Karnofsky scale ≥70%. Patients are administered radiotherapy with or without chemotherapy with a curative intent and signed the informed consent form: intervention (I) and comparison (C): The treatment group is assigned to receive oral zinc sulfate, patients in the control group are asked to take placebo capsules or other active drugs that were similar in shape, taste, and color to the zinc sulfate capsule; outcomes (O): The primary outcome measures include the incidence and severity of oropharyngeal mucositis, and the secondary outcomes are the onset of oropharyngeal mucositis; and study design (S): In this meta-analysis, only randomized controlled trials (RCTs) are eligible. If an abstract has a sufficient data, it will be also considered. It will be considered when the language of included studies is English or Chinese.

The study will be excluded if it meets at least one of the following criteria: previously administration of cytotoxic chemotherapy or radiotherapy, infection of mouth and systemic infection, previous oropharyngeal mucositis, duplication with poor methodology, and insufficient data.

### 2.2 Definition of outcomes

In this meta-analysis, incidence of oropharyngeal mucositis is defined as the value of number of experienced the oropharyngeal mucositis irrespective of grade divided by the total number of cancer patients completed the whole study. The severity of oropharyngeal mucositis is graded according to the Radiation Therapy Oncology Group scoring system or the oropharyngeal mucositis assessment scale (OMAS).^[24,25]^ Onset of oropharyngeal mucositis was considered as the time of definitively diagnosed oropharyngeal mucositis

### 2.3 Identification of eligible literature

**Figure 2:**
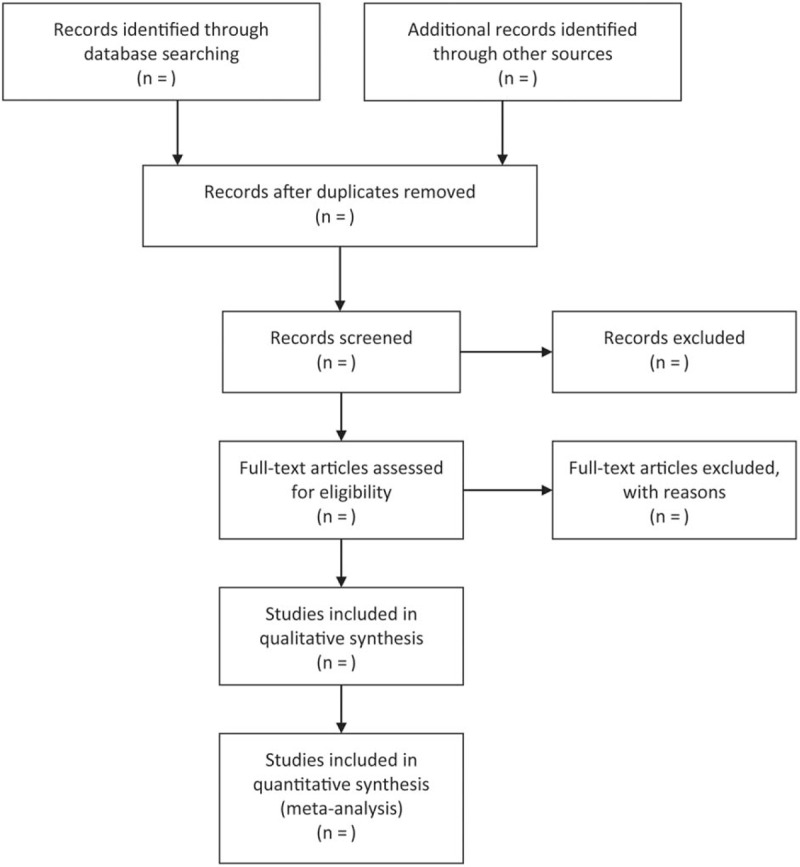
The PRISMA flow chart to depict the process of searching and screening citations.

The PRISMA flow chat will be used to depict the process of searching and screening citations (Fig. 2).^[26]^ Relevant information will be identified by a comprehensive search of the following electronic databases: PubMed, Cochrane Central Register of Controlled Trials (CENTRAL), Embase, and EBSCO from their inception to October 2018. Reference lists of all included full-text articles and topic related reviews will be hand-searched for additional original publications. Meanwhile, the Clinicaltrial.gov will be electronically retrieved for the purpose of covering all potential eligible studies. The literature search strategy for databases is developed by the lead author (TS) and an experienced systematic reviewer (XT), using a combination of Medical Subject Headings (MeSH) and keyword terms. The “zinc,” “oropharyngeal mucositis,” and “random” will be the main terms to construct search algorithms. A draft literature search strategy for Pubmed database is presented in Table 1. The search strategy will be revised for every database considering the difference among these databases. Endnote X7.0 reference management software package will be used to manage all the search results during the review period.

**Table 1:**
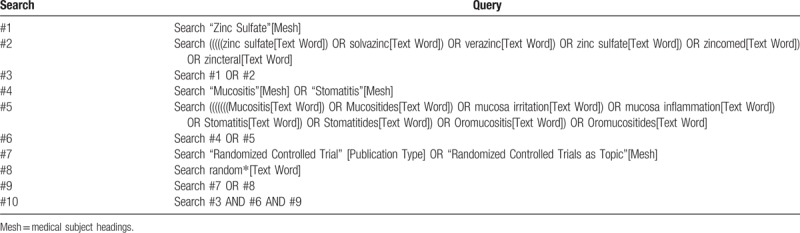
PubMed search algorithms.

### 2.4 Data extraction

Firstly, LJY and HC review the titles and abstracts to assess the eligibility. Then, when the articles appear to meet included criteria, they will be retrieved in full and independently considered for inclusion by the 2 members. The third author (XEL) will resolve disagreements in opinion of studies for inclusion and the reasons for excluded studies will be recorded.

Data from included full-text articles will be extracted by 2 reviewers (WQC and LJY). The basic information and data for the specific outcomes, such as first author, publication year, age of participants, sample size, details of interventions, and outcomes of interest, are extracted on the basis of the standard data extraction form designed by XT which was used in our previous systematic reviews and meta-analyses (seen as supplemental Table). Disagreements about the extraction of the basic information and data will be resolved through discussion.

### 2.5 Quality assessment of individual study

The 2 independent reviewers are assigned to evaluate the risk of bias from 7 domains, including randomization sequence generation, allocation concealment, blinding of participants, blinding of study personnel, blinding of outcome assessors, incomplete outcome data, selective reporting and other bias with the Cochrane risk of bias assessment tool.^[27]^ A study will be assigned a risk level of “high risk of bias,” “unclear risk of bias,” or “low risk of bias” according to the match level between the actual information and the evaluation criteria. Any discrepancies between the 2 reviewers will be resolved through 3rd-party adjudication.

### 2.6 Statistical analysis

Meta-analysis will be performed when the identified studies are comparable. To calculate the pooled effects, the continuous data will be recorded as mean difference (MD) with 95% confidence intervals (CIs) and the dichotomous data will be extracted as relative risk (RR) with 95% CIs. At the same time, traditional pairwise meta-analysis will be conducted based on the random-effect model, which incorporates within and between studies heterogeneity, to estimate the summarized RR and 95% CIs.^[28]^ The Chi-squared method and the *I*^2^ statistic will be adopted to test the heterogeneity and the proportion of the overall variation that is attributable to between study heterogeneity.^[29,30]^ All analyses will be conducted using the RevMan 5.3 (The Nordic Cochrane Centre, The Cochrane Collaboration, 2013, Copenhagen, Denmark) and Stata 12 (StataCorp, College Station, TX).

Given possible important heterogeneity among the included studies, subgroup analyses and meta-regression analyses are perhaps used to explore the possible sources. Subgroup analyses are planned in accordance with chemoradiotherapy and assessment scales of oropharyngeal mucositis. Sensitivity analyses may be used by analyzing only studies considered at low risk of bias. When the number of studies in the meta-analysis is more than 10, the funnel plot is necessary to be performed for identifying publication bias.^[31]^

### 2.7 Subgroup and sensitivity analyses

In the case of possible important heterogeneity, we will explore the possible sources using subgroup and meta-regression analyses. Subgroup analyses are planned for dose of radiotherapy and control regimes. Sensitivity analyses are planned for prevention of radiotherapy-induced oropharyngeal mucositis by analyzing only studies considered at low risk of bias.

### 2.8 Publication bias

For single outcome, we will draw the funnel plot to identify publication bias if the number of studies analyzed is more than 10.^[32]^ Moreover, we will also perform the Egger linear regression test to quantitatively detect the symmetric or a symmetric of funnel plot.^[32]^

## 3 Discussions

The definition of head and neck cancer has not yet been identified all around the world. In this study, we will adopt the definition of head and neck cancer which was defined by the International Agency for Research on Cancer (IRAC) in 2014.^[1]^ Within the head and neck, the major anatomical sites involve the nasal cavity, nasopharynx, oral cavity, oropharynx, larynx, and hypopharynx. With rapid population growth and aging worldwide, the rising prominence of head and neck cancer has been an important public health problem. Radiotherapy with or without chemotherapy has been the primary option in the treatment of head and neck cancer. However, radiotherapy inevitably brings about short- and long-term side effects because of the cytotoxic effect. Oropharyngeal mucositis is considered as one of the most stressful side effects of head and neck irradiation. Mucositis-associated pain significantly impairs oral functions such as deglutition and taste, resulting in the nutritional problems. The severe oropharyngeal mucositis can decrease the quality of sleep, and disturb phonation and aspiration. Besides, severe oropharyngeal mucositis also increases the risk of infections and the hospitalization.^[33]^ Hence, it is worthwhile to find effective intervention to prevent or minimize oropharyngeal mucositis during the course of radiotherapy.

Although published studies have come up with plenty of different methods to prevent and treat radiation-induced oropharyngeal mucositis, the effect of these interventions has not been completely identified and the prophylaxis of oropharyngeal mucositis is still an unsolved problem. Published evidences exactly demonstrated that zinc is the important element for multiple cellular activities and the regulation of immune system. Moreover, zinc works as an organelle stabilizer and a stabilizer of the structure of DNA, RNA, and ribosome, which can accelerate wound healing.^[15,19]^ So some researchers tried to confirm the benefit of zinc in preventing radiation-induced mucositis in the head and neck cancer. To date, 4 studies explored the benefit but the different results were published. Three studies showed zinc could reduce the incidence and severity of mucositis in patients with head and neck cancer undergoing radiotherapy with or without chemotherapy.^[9,20,22]^ But Gorgu et al found that zinc sulfate prophylaxis did not reduce incidence of mucositis.^[21]^ Therefore, the efficacy of zinc to prevent radiation-induced oropharyngeal mucositis remains controversial. Although several meta analyses about cancer treatment-induced oropharyngeal mucositis were published, these articles did not include the complete studies and perform the subgroup to clearly clarify the effect for prophylaxis with oral zinc sulfate against radiation-induced oropharyngeal mucositis in patients with head and neck cancer.^[34,35]^

The present study will be the first systematic review and meta-analysis to elucidate current evidence of the efficacy of zinc to prevent radiation-induced oropharyngeal mucositis in the head and neck cancer. It will highlight that further exploration is needed to inform recommendations of radiation-induced oropharyngeal mucositis.

### 3.1 Ethics and dissemination

Ethics approval and patient written informed consent will not be required because all analyses in the present study will be performed based on data from published studies. The systematic review and meta-analysis will be submitted to a peer reviewed scientific journal for publication.

## Author contributions

Ting Shuai, Xu Tian, and Xiu-E Li conceived the study and developed the study criteria. Li-juan Yi and Hui Chen searched the literature and Xu Tian designed the data extraction form. Ting Shuai wrote the protocol. Xiu-E Li and Wei-Qing Chen revised the manuscript. All authors have read, and approved the final manuscript.

**Conceptualization:** Ting Shuai, Xu Tian.

**Data curation:** Ting Shuai, Li-Juan Yi, Hui Chen.

**Formal analysis:** Ting Shuai, Hui Chen.

**Investigation:** Li-Juan Yi, Xu Tian.

**Methodology:** Ting Shuai, Li-Juan Yi, Xu Tian.

**Resources:** Hui Chen.

**Supervision:** Xiu-E Li.

**Validation:** Xiu-E Li.

**Writing – original draft:** Ting Shuai, Xu Tian.

**Writing – review & editing:** Wei-Qing Chen, Xiu-E Li.

## Supplementary Material

Supplemental Digital Content
